# Delaware Dental Association

**Published:** 1867-06

**Authors:** Samuel Marshall


### DELAWARE DENTAL ASSOCIATION.

The Seventh Semi-Annual Convention of the "Delaware Dental Association," will assemble at Dover, Delaware, on Thursday, the 13th day of June at 10 o'clock A. M., at which time we hope and expect to be greeted by your presence.

Business of the most vital interest to our profession will claim the attention of this meeting; therefore, it is important that every Dentist in this region should, if necessary, make some sacrifice to be present.

A Bill to regulate the practice, and, to some extent, control the members of our profession in Delaware, will be considered and prepared to lay before the next Legislature, of the State.

The Constitution and By-Laws of our Association will be ready for distribution, and other business of interest and importance to the profession will be transacted. Come and help us, or abide the consequences.

The members from Wilmington and vicinity, will go to Dover the evening before the meeting, on the train, which, under the present arrangement, leaves Wilmington at 6 P. M., and return the next evening.

A change of hour is anticipated, but that will not materially change the programme. All who can go down and spend a social evening before the meeting, will no doubt enjoy it.

Professionally, yours,

SAMUEL MARSHALL,

MAY, 1867. *Corresponding Secretary.*

